# Association between intraoperative hyperglycemia/hyperlactatemia and acute kidney injury following on-pump cardiac surgery: a retrospective cohort study

**DOI:** 10.3389/fcvm.2023.1218127

**Published:** 2023-12-08

**Authors:** Qiyu He, Zhimin Tan, Dongxu Chen, Shuang Cai, Leng Zhou

**Affiliations:** ^1^ Department of Urology, West China Hospital of Sichuan University, Chengdu, Sichuan Province, China; ^2^ Department of Anesthesiology, West China Hospital of Sichuan University, Chengdu, Sichuan Province, China; ^3^ Department of Anesthesiology, West China Second Hospital of Sichuan University, Chengdu, Sichuan Province, China; ^4^ Key Laboratory of Birth Defects and Related Diseases of Women and Children (Sichuan University), Ministry of Education, Chengdu, Sichuan Province, China

**Keywords:** acute kidney injury, cardiac surgery, hyperglycemia, hyperlactatemia, intraoperative

## Abstract

**Background:**

Despite the long-lasting notion about the substantial contribution of intraoperative un-stabilization of homeostasis factors on the incidence on acute kidney injury (AKI), the possible influence of intraoperative glucose or lactate management, as a modifiable factor, on the development of AKI remains inconclusive.

**Objectives:**

To investigated the relationship between intraoperative hyperglycemia, hyperlactatemia, and postoperative AKI in cardiac surgery.

**Methods:**

A retrospective cohort study was conducted among 4,435 adult patients who underwent on-pump cardiac surgery from July 2019 to March 2022. Intraoperative hyperglycemia and hyperlactatemia were defined as blood glucose levels >10 mmol/L and lactate levels >2 mmol/L, respectively. The primary outcome was the incidence of AKI. All statistical analyses, including *t* tests, Wilcoxon rank sum tests, chi-square tests, Fisher's exact test, Kolmogorov-Smirnov test, logistic regression models, subgroup analyses, collinearity analysis, and receiver operating characteristic analysis, were performed using the statistical software program R version 4.1.1.

**Results:**

Among the 4,435 patients in the ﬁnal analysis, a total of 734 (16.55%) patients developed AKI after on-pump cardiac surgery. All studied intraoperative metabolic disorders was associated with increased AKI risk, with most pronounced odds ratio (OR) noted for both hyperglycemia and hyperlactatemia were present intraoperatively [adjusted OR 3.69, 95% confidence intervals (CI) 2.68–5.13, *p* < 0.001]. Even when hyperglycemia or hyperlactatemia was present alone, the risk of postoperative AKI remained elevated (adjusted OR 1.97, 95% CI 1.50–2.60, *p* < 0.001).

**Conclusion:**

The presence of intraoperative hyperglycemia and hyperlactatemia may be associated with postoperative acute kidney injury (AKI) in patients undergoing on-pump cardiac surgery. Proper and timely interventions for these metabolic disorders are crucially important in mitigating the risk of AKI.

## 1. Introduction

Each year, approximately two million patients worldwide undergo cardiac surgery with supported by cardiopulmonary bypass (CPB), with up to 40% of them develop postoperative acute kidney injury (AKI) (1–3). AKI is a prevalent condition and has the potential to prolong hospitalization, promote the progression to chronic kidney diseases (CKD), and increase in-hospital mortality by five-fold (4, 5). Previous studies have identified several factors that contribute to the incidence of AKI after cardiac surgery. While some risk factors cannot be modified, such as advanced age (6), increased body mass index (BMI) (7), female (8), smoking status (8), and preoperative renal dysfunction (9), other modifiable factors warrant attention to minimize their impact.

Glucose and lactate are essential components of human metabolism, intricately involved in various physiological and pathological metabolic pathways. Hyperglycemia and hyperlactatemia are frequently observed in cardiac surgery (10). However, the potential influence of perioperative glucose and lactate concentrations on the development of AKI as modifiable factors remains largely inconclusive. For instance, study have demonstrated a significant correlation between the level of perioperative glucose control and postoperative renal insufficiency in non-diabetic patients underwent cardiac surgery, but such association does not be found in diabetic patients (11). Moreover, interventional studies based on perioperative glucose management observed a reduced risk of postoperative AKI (12), which implies an important role of preoperative psychological factors on AKI and its related conditions. Besides, previous studies also have emphasized the importance of early identification of hyperlactatemia and have proposed that early postoperative hyperlactatemia is a well-established predictor of mortality than late postoperative hyperlactatemia (13). Methodological shortcomings of most existing investigations included small sample sizes (14), limited type of cardiac surgery (15), limited control for confounders (16), and varied definitions of AKI (11, 17). Consequently, leaving the actual role of metabolic factors (specific intraoperative hyperglycemia/hyperlactatemia) on the risk of AKI largely unclear.

In this retrospective observational cohort study, we aimed to investigate the relationship between intraoperative both hyperglycemia and hyperlactatemia or any of them and the incidence of postoperative AKI in patients underwent on-pump cardiac surgery.

## 2. Materials and methods

### 2.1. Study design

Retrospective data were collected from patients admitted to West China Hospital of Sichuan University. Two independent investigators retrieved information according to data documented in the anesthesia information management system (AIMS) and electronic medical record (EMR) system by using a standardized data collection form. Strengthening the Reporting of Observational Studies in Epidemiology (STROBE) guidelines were followed to guarantee the study quality (18). The institutional review boards at West China Hospital of Sichuan University approved this retrospective observational study (No. 869/2021, July 23, 2021) with a waiver of informed consent. The analysis plan was drafted after completion of data collection.

### 2.2. Inclusion and exclusion criteria

**Figure 1:**
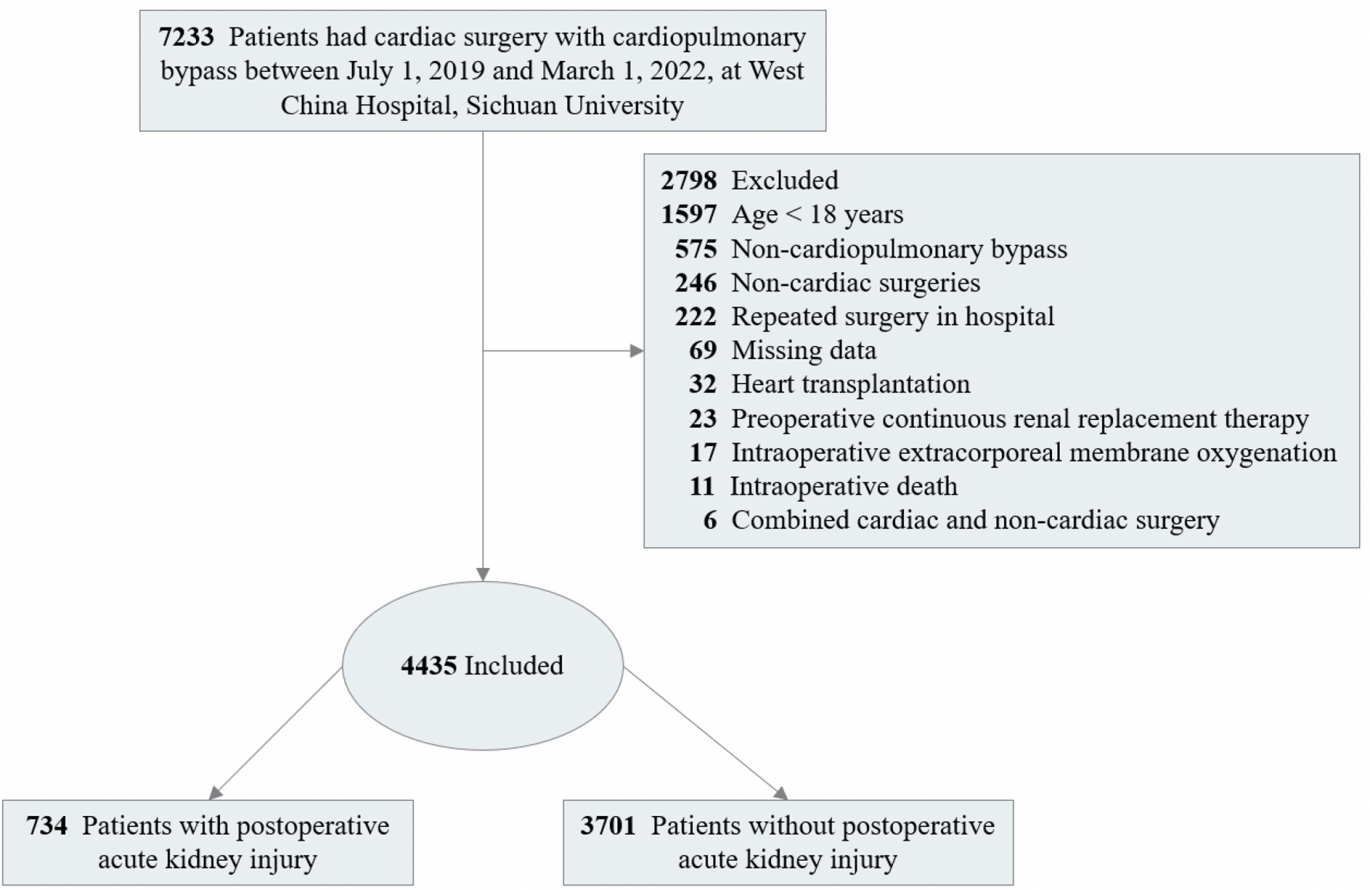
Flow chart of patient enrollment.

The study included adult patients (≥18 years) who underwent cardiac surgery with CPB, including the combination of multiple cardiac surgical procedures, such as valve surgery conducted on its own or together with other types of surgery (e.g., the Bentall procedure, removal of atrial thrombus, or myxoma resection), at West China Hospital, Sichuan University from July 2019 to March 2022. Patients with baseline serum creatinine concentration (Scr) values exceeding 4.0 mg/dl (353.6 μmol/L) or those already receiving renal dialysis were excluded due to their poor baseline renal function, which prevented further kidney injury assessment. Meanwhile, patients who underwent heart transplantation, combined non-cardiac surgery (e.g., radical lung cancer surgery, thymic tumor resection, and mediastinal tumor resection), received intraoperative extracorporeal membrane oxygenation (ECMO) for weaning off CPB in the operating room, or died in operation were excluded from the study (Figure 1). When patients underwent repeated cardiac procedures during the study period, only the first procedure was enrolled.

### 2.3. Exposure and outcome

Data collected encompassed demographic information, comorbidities, surgical-related and anesthesia-related condition, perioperative medications, and laboratory examination. Among them, comorbidities were determined through a rigorous review of Electronic Health Records and assessed quantitatively using the Age-adjusted Charlson index (ACCI) and European System for Cardiac Operative Risk Evaluation II (EuroSCORE II) (19).

Intraoperative glycemia and lacticaemia were retrieved from electronic AIMS systems. Each timepoint during surgery, including the time of patient admission to the operating room, the start and end of CPB, and the time of patient discharge from the operating room, measured by automatic blood gas and electrolytes analyzer (Cobas B 123, Roche Ltd., Basel, Switzerland). Any instance of blood glucose concentration exceeding 10 mmol/L (20) or blood lactate level greater than 2 mmol/L (21) is considered as occurrence intraoperative hyperglycemia or hyperlactatemia. In present study, at minimum, patients needed to have a baseline arterial blood gas measurement and another one measurement during surgery to be included in our study. As Lactate and glucose levels may have an interdependent relationship in the context of postoperative AKI, and only a small number of patients tested positive for a single exposure factor. We set the exposure to two scenarios: hyperglycemia and hyperlactatemia; hyperglycemia or hyperlactatemia.

Postoperative AKI was confirmed by the Kidney Disease: Improving Global Outcomes (KDIGO) criteria (22). As data on urine output was not available, it was not considered as an alternative definition for AKI. In fact, Scr is a generally accepted and adequate parameter for defining AKI (23). Consequently, postoperative AKI was defined as an increase in Scr by ≥0.3 mg/dl (≥26.5 µmol/L) within 48 h, or an increase of at least 1.5 times the baseline Scr in the 7 days after surgery. We also categorized AKI by KDIGO stage as follows: stage 1 AKI, creatinine rise 0.3 mg/dl or greater within 48 h or 1.5–1.9 times baseline within first 7-day after surgery; stage 2 AKI, creatinine rise 2.0–2.9 times baseline within 7-day after surgery; and stage 3 AKI, creatinine rise to 4.0 mg/dl or greater or 3.0 times baseline. Keeping with the KDIGO criteria, we limit our evaluation period to 7 days post-operation rather than considering serum creatinine measurements throughout the hospital stay.

### 2.4. Statistical analysis

Descriptive statistics were computed as mean (standard deviation, SD) or median (25th, 75th percentiles) for continuous variables and frequencies with percentages for categorical variables. The Kolmogorov-Smirnov test was used for normality testing. Continuous (normal and nonnormal) and categorical measures were appropriately tested with *t* tests, Wilcoxon rank sum tests, chi-square tests, or Fisher's exact test.

We investigated the relationship between intraoperative hyperglycemia and hyperlactatemia and the incidence of AKI using logistic regression models to derive odds ratios (ORs) with 95% confidence intervals (CIs). Covariates were identiﬁed through literature reviews. We employed the variance inflation factor (VIF) to assess the presence of multicollinearity among the independent variables (Supplementary Table S1). The VIF values for all variables were found to be less than 10, indicating that the assumption of multicollinearity is not violated (24). Models were partly (models 1–4) or fully (model 5) adjusted for demographic characteristics (i.e., age, sex, and BMI), preoperative comorbidities and renal function [i.e., ACCI, EuroSCORE Ⅱ, New York Heart Association Classification (NYHA), previous cardiac surgery, preoperative Cystatin C (Cys-C), preoperative estimated glomerular filtration rate (eGFR), and preoperative Scr], operative data (i.e., operation type, anesthesia method, CPB duration, operation duration, emergent surgery), intraoperative fluid management [i.e., red blood cell (RBC), plasma, cryoprecipitate, platelet, crystalloid, and colloid infusion, and autotransfusion], and intraoperative vasoactive agents (i.e., epinephrine, norepinephrine, million, dopamine, and nitroglycerin infusion). To detect the modification effect of some important factors, we performed subgroup analyses by age group (≤50 or >50 years), sex (male or female), ACCI (0, 1, 2, ≥3), preoperative Cys-C (≤1.4 or >1.4 mg/L), preoperative eGFR (≤90 or >90 ml/min/1.73 m^2^), preoperative Scr (≤133 or >133 µmol/L), emergent surgery (yes or no), operation length (≤4 or >4 h), and CPB duration (≤2 or >2 h). The rationale and the chosen of breakpoints for dividing into different subgroups were based on previous studies and considerations of subgroup sample size balance (25). Variables such as gender and age are commonly used as sub-classification factors. The ACCI is a widely applied system for assessing comorbidities. Meanwhile, Cys-C, Scr, and eGFR are commonly utilized indicators to assess impaired kidney function. The differences in ORs were assessed by introducing an interaction term to the logistic models or by Wald test. During the peer-review process, we conducted a receiver operating characteristic (ROC) analysis on various models, in accordance with the recommendations provided by the reviewers.

All statistical analyses were performed using the statistical software program R version 4.1.1 (The R Foundation for Statistical Computing, https://www.r-project.org/). Statistical significance was defined as 2-sided *p* < 0.05.

### 2.5. Sample size considerations

No statistical power calculation was conducted before the study and the sample size was based on the available data. We performed a *post hoc* analysis to assess the effect size we could detect based on actual data. Only 24.87% of patients had not experienced hyperglycemia or hyperlactatemia intraoperatively, and the incidence of AKI was estimated to be 16.55%. We could detect an OR of AKI greater than 1.97 associated with intraoperative hyperglycemia and hyperlactatemia with a type I error of 0.05 using our current sample size.

## 3. Results

### 3.1. Patient and surgery characteristics

Among the 7,233 patients underwent cardiac surgery screened in the cohort, a total of 2,798 patients were excluded, of whom 1,697 aged less than 18 years old, 625 received non-CPB surgeries, 272 received repeated cardiac surgery, 69 with data missing, 53 had preoperative Scr > 353.6 μmol/L, 32 received heart transplantation, 27 received combined cardiac and non-cardiac surgery, 17 used ECMO, and 6 died in the operating room. As shown in Figure 1, 4,435 surgeries were included in analysis.

The median age of the patients was 53 years [interquartile range (IQR): 46–61], and 50.03% (2,219 of 4,435, Table 1) of the patients were male. 258 (5.82%) patients had a history of diabetes and 7.51% (333/4,435) of patients had previous cardiac surgery. 1,335 (30.55%) underwent heart valve related surgery, 266 (6%) underwent coronary artery bypass grafting (CABG). In present study, 8.70% of patients (386/4,435) received an emergency procedure.

**Table 1:** Demographics and clinical characteristics stratified by primary outcome.

	Total	Postoperative non-AKI	Postoperative AKI	*p*-value
No. of patients	4,435	3,701	734	
Demographic characteristics				
Age, years, median (IQR)	53.00 (46.00, 61.00)	53.00 (45.00, 60.00)	55.00 (48.00, 64.00)	<0.001^e^
Sex, *n* (%)				<0.001^f^
Male	2,219 (50.03)	1,717 (46.39)	502 (68.39)	
Female	2,216 (49.97)	1,984 (53.61)	232 (31.61)	
BMI, kg/m^2^, median (IQR)	23.24 (21.15, 25.54)	23.14 (21.08, 25.33)	24.00 (21.64, 26.57)	<0.001^e^
Preoperative comorbidities				
ACCI, *n* (%)				<0.001^f^
0	1,237 (27.89)	1,091 (29.48)	146 (19.89)	
1	1,347 (30.37)	1,146 (30.96)	201 (27.38)	
2	986 (22.23)	814 (21.99)	172 (23.43)	
≥3	865 (19.50)	650 (17.56)	215 (29.29)	
ASA classification, *n* (%)				<0.001^f^
<3	3,986 (89.88)	3,414 (92.25)	572 (77.93)	
≥3	449 (10.12)	287 (7.75)	162 (22.07)	
NYHA, *n* (%)				<0.001^f^
1	299 (6.74)	262 (7.08)	37 (5.04)	
2	2,467 (55.63)	2,089 (56.44)	378 (51.50)	
3	1,572 (35.45)	1,279 (34.56)	293 (39.92)	
4	97 (2.19)	71 (1.92)	26 (3.54)	
EuroSCORE II, median (IQR)	2.00 (1.00, 4.00)	2.00 (1.00, 4.00)	3.00 (1.00, 6.00)	<0.001^e^
LVEF, *n* (%)				0.008^f^
≥50%	4,003 (90.26)	3,362 (90.84)	641 (87.33)	
30%–49%	403 (9.09)	314 (8.48)	89 (12.13)	
<30%	29 (0.65)	25 (0.68)	4 (0.54)	
Diabetes mellitus, *n* (%)	258 (5.82)	194 (5.24)	64 (8.72)	<0.001^f^
CHD, *n* (%)	374 (8.43)	289 (7.81)	85 (11.58)	0.001^f^
COPD, *n* (%)	201 (4.53)	156 (4.22)	45 (6.13)	0.029^f^
Unstable angina, *n* (%)	76 (1.71)	59 (1.59)	17 (2.32)	0.222^f^
Recent myocardial infarct^a^, *n* (%)	64 (1.44)	40 (1.08)	24 (3.27)	<0.001^f^
Pulmonary hypertension^b^, *n* (%)				0.351^f^
None	4,175 (94.14)	3,490 (94.30)	685 (93.32)	
Moderate	216 (4.87)	173 (4.67)	43 (5.86)	
Severe	44 (0.99)	38 (1.03)	6 (0.82)	
Previous cardiac surgery, *n* (%)	333 (7.51)	262 (7.08)	71 (9.67)	0.018^f^
Operative data				
Emergency, *n* (%)	386 (8.70)	245 (6.62)	141 (19.21)	<0.001^f^
Anesthesia, *n* (%)				0.360^f^
Intravenous and inhalation	3,824 (86.22)	3,186 (86.08)	638 (86.92)	
Total intravenous	405 (9.13)	347 (9.38)	58 (7.90)	
Inhalation	206 (4.64)	168 (4.54)	38 (5.18)	
Operation duration, min, median (IQR)	248.00 (208.00, 307.50)	240.00 (204.00, 292.00)	310.00 (241.25, 407.75)	<0.001^e^
CPB duration, min, median (IQR)	123.00 (92.00, 163.00)	117.00 (89.00, 152.00)	164.00 (120.00, 210.00)	<0.001^e^
Operation type, *n* (%)				<0.001^f^
CABG	266 (6.00)	205 (5.54)	61 (8.31)	
CHD	291 (6.56)	281 (7.59)	10 (1.36)	
Single valve	1,157 (26.09)	980 (26.48)	177 (24.11)	
Multi valve	198 (4.46)	159 (4.30)	39 (5.31)	
Vascular	603 (13.60)	417 (11.27)	186 (25.34)	
Mixed^c^	1,594 (35.94)	1,363 (36.83)	231 (31.47)	
Others^d^	326 (7.35)	296 (8.00)	30 (4.09)	
Preoperative laboratory examination				
Preoperative HB, g/dl, median (IQR)	135.00 (123.00, 147.00)	135.00 (123.00, 147.00)	133.00 (119.00, 145.00)	<0.001^e^
Preoperative NT-pro BNP, pg/ml, median (IQR)	506.00 (133.00, 1,377.00)	456.00 (122.00, 1,261.50)	821.50 (259.00, 2,235.75)	<0.001^e^
Preoperative cTnT, ug/L, median (IQR)	10.50 (7.10, 17.48)	9.90 (6.60, 15.90)	15.65 (10.00, 28.28)	<0.001^e^
Preoperative Cys C, mg/L, median (IQR)	0.99 (0.87, 1.14)	0.97 (0.86, 1.11)	1.09 (0.95, 1.32)	<0.001^e^
Preoperative eGFR, ml/min/1.73 m^2^, median (IQR)	90.36 (75.87, 101.72)	91.42 (77.81, 102.55)	84.22 (65.36, 96.38)	<0.001^e^
Preoperative albumin, g/L, median (IQR)	43.00 (40.30, 45.70)	43.25 (40.70, 45.90)	41.80 (38.60, 44.30)	<0.001^e^
Preoperative Scr, µmol/L, median (IQR)	76.00 (64.00, 89.00)	74.00 (64.00, 86.00)	84.00 (71.00, 102.00)	<0.001^e^
Intraoperative fluid management				
RBC infusion, *n* (%)	463 (10.44)	317 (8.57)	146 (19.89)	<0.001^f^
Plasma infusion, *n* (%)	429 (9.67)	287 (7.75)	142 (19.35)	<0.001^f^
Cryoprecipitate infusion, *n* (%)	24 (0.54)	18 (0.49)	6 (0.82)	0.269^f^
Platelet infusion, *n* (%)	1,376 (31.03)	976 (26.37)	400 (54.50)	<0.001^f^
Autotransfusion, ml, median (IQR)	300.00 (250.00, 500.00)	300.00 (250.00, 500.00)	400.00 (300.00, 600.00)	<0.001^e^
Crystalloid, ml, median (IQR)	700.00 (500.00, 1,000.00)	700.00 (500.00, 1,000.00)	800.00 (550.00, 1,100.00)	<0.001^e^
Colloid, ml, median (IQR)	0.00 (0.00, 100.00)	0.00 (0.00, 50.00)	0.00 (0.00, 300.00)	<0.001^e^
Urinary, ml, median (IQR)	900.00 (550.00, 1,300.00)	950.00 (600.00, 1,400.00)	800.00 (500.00, 1,200.00)	<0.001^e^
Intraoperative vasoactive agents				
Epinephrine, *n* (%)	3,881 (87.51)	3,207 (86.65)	674 (91.83)	<0.001^f^
Total epinephrine, μg, median (IQR)	178.10 (56.35, 308.50)	164.50 (49.90, 283.00)	267.30 (109.25, 432.90)	<0.001^e^
Norepinephrine, *n* (%)	1,155 (26.04)	856 (23.13)	299 (40.74)	<0.001^f^
Total norepinephrine, μg, median (IQR)	0.00 (0.00, 0.00)	0.00 (0.00, 0.00)	0.00 (0.00, 0.20)	<0.001^e^
Millinon, *n* (%)	458 (10.33)	329 (8.89)	129 (17.57)	<0.001^f^
Total millinon, mg, median (IQR)	0.00 (0.00, 0.00)	0.00 (0.00, 0.00)	0.00 (0.00, 0.00)	<0.001^e^
Dopamine, *n* (%)	21 (0.47)	19 (0.51)	2 (0.27)	0.560^f^
Total dopamine, mg, median (IQR)	0.00 (0.00, 0.00)	0.00 (0.00, 0.00)	0.00 (0.00, 0.00)	0.385^e^
Nitroglycerin, *n* (%)	3,082 (69.49)	2,603 (70.33)	479 (65.26)	0.007^f^
Total nitroglycerin, mg, median (IQR)	897.00 (0.00, 3,114.80)	955.50 (0.00, 3,120.00)	594.85 (0.00, 2,999.00)	0.062^e^
Coronary heart disease related drugs				
Aspirin, *n* (%)	181 (4.82)	132 (4.28)	49 (7.26)	0.001^f^
β-blockers, *n* (%)	607 (13.69)	462 (12.48)	145 (19.75)	<0.001^f^
LMWH, *n* (%)	251 (5.66)	198 (5.35)	53 (7.22)	0.055^f^

Data are present in *n* (%), mean (SD) or median (IQR).

ACCI, age-adjusted Charlson comorbidity index; ASA, American Society of Anesthesiologists classification; BMI, body-mass index; CABG, coronary artery bypass grafting; CHD, congenital heart disease; COPD, chronic obstructive pulmonary disease; CPB, cardiopulmonary bypass; cTnT, cardiac troponin T; Cys C, cystatin C; EuroSCORE II, European system for cardiac operative risk evaluation II; eGFR, estimated glomerular filtration rate; HB, hemoglobin; IQR, interquartile range; LVEF, left ventricular ejection fractions; LMWH, low molecular weight heparin; NT-pro BNP, N-terminal pro-brain natriuretic peptide; NYHA, New York Heart Association Classification; RBC, red blood cell; SD, standard deviation; Scr, serum creatinine concentration.

^a^ Recent myocardial infarction is defined as any diagnosed myocardial infarction in past 90 days before the operation.

^b^ Moderate pulmonary hypertension is defined as pulmonary artery systolic pressure 31–55 mmHg, and severe pulmonary hypertension as more than 55 mmHg.

^c^ Mixed operations are referred to operations combined with CABG, and/or valve, and/or CHD, and/or others.

^d^ Others operations are referred to operations that could not be included in the types above (i.e., cardiac myxoma, pulmonary endarterectomy, etc.).

^e^ *p*-values are derived from: *u*-test.

^f^ *p*-values are derived from: chi-square test or Fisher exact test.

A total of 734 (16.55%) patients developed AKI after cardiac surgery with CPB. Table 1 summarizes the baseline characteristics of patients stratified by postoperative AKI. By the presence of AKI, we found patients with AKI were more likely to be older [55 years (IQR: 48–64) vs. 53 years (IQR: 45–60)], male (68.39% vs. 31.61%), having higher BMI [24.00 (IQR: 21.64–26.57) vs. 23.14 (IQR: 21.08–25.33)] and higher possibilities of receiving emergent procedures (19.21% vs. 6.62%). Also, AKI patients tended to have more severe indication diseases, presenting as having higher EuroSCORE II scores [3 (IQR: 1–6) vs. 2 (IQR: 1–4)], longer operation [310 min (IQR: 241.25–407.75) vs. 240 min (IQR: 204.00–292.00)] and long-last CPB duration [164 min (IQR: 120–210) vs. 117 min (IQR: 89–112)] than patients without AKI. In general, patients with AKI exhibited a poorer health status.

**Figure 2:**
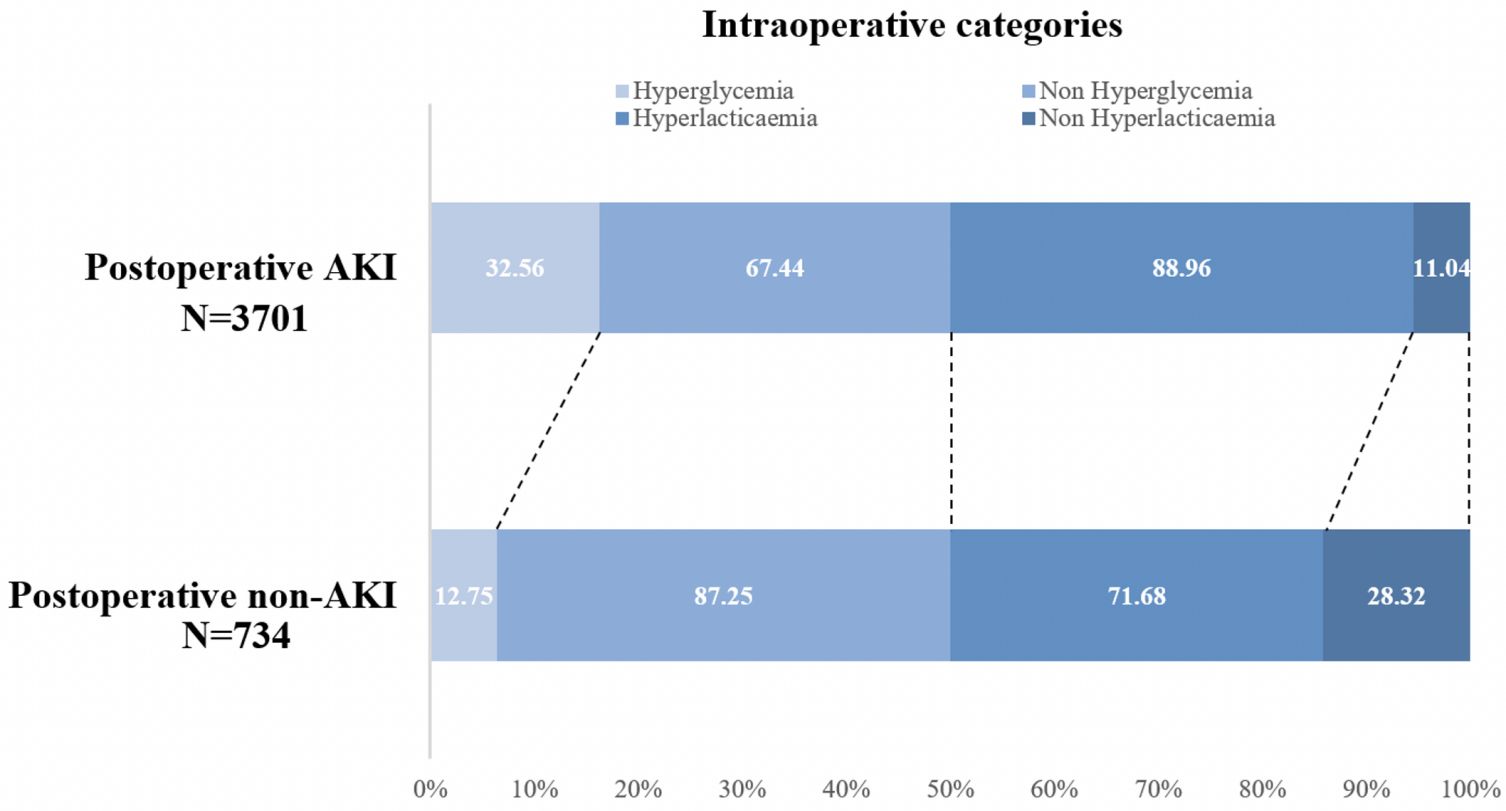
Intraoperative hyperglycemia/hyperlactatemia categories in the postoperative AKI group (*n* = 734) and postoperative non-AKI (*n* = 3,701) group. AKI, acute kidney injury.

Among the 734 patients who developed AKI postoperatively, 235 (34.31% of 685) had both intraoperative hyperglycemia and hyperlactatemia, 422 (15.94% of 2,647) had either intraoperative hyperglycemia or hyperlactatemia, and only 77 (6.98% of 1,103) had neither intraoperative hyperglycemia nor hyperlactatemia. In contrast, among those who did not develop AKI, only 12.16% had both intraoperative hyperglycemia and hyperlactatemia (Figure 2). The distributions of AKI incidence by the presence of intraoperative hyperglycemia and/or hyperlactatemia was presented in Supplementary Table S2.

### 3.2. Association between both intraoperative hyperglycemia and hyperlactatemia or any of them and postoperative AKI

The association between both hyperglycemia and hyperlactatemia or any of them intraoperatively and AKI was significant after partial or full adjustment for confounders listed in Supplementary Table S3.

**Figure 3:**
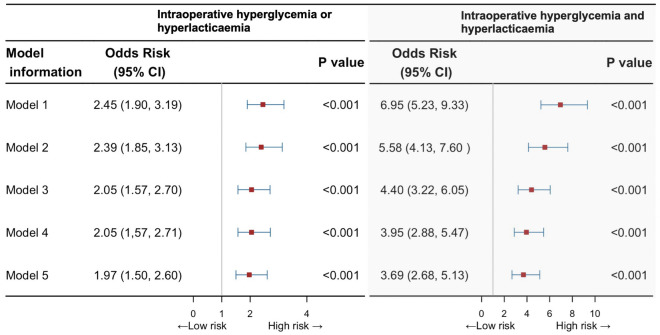
Risk of postoperative acute kidney injury with intraoperative both hyperglycemia and hyperlactatemia, or any of them, compared with unexposed individuals. CI, confidence interval.

The multivariable analysis showed the intraoperative either hyperglycemia or hyperlactatemia corresponded to an OR of 2.45 (95% CI, 1.90–3.19) adjusted for age, sex, and BMI (model 1), which in sequence decreased to 2.39 (95% CI, 1.85–3.31), 2.05 (95% CI, 1.57–2.70), 2.05 (95% CI, 1.57–2.71) when adjusting for more confounders: preoperative comorbidities and renal function (model 2), operative data (model 3) and intraoperative fluid management (model 4), and then decreased to 1.97 (95% CI, 1.50–2.60) when adding intraoperative vasoactive agents into the models (model 5; Figure 3). Similarly, these proportions in the group of people with hyperglycemia and hyperlactatemia corresponded to an age-, sex-, and BMI-adjusted OR of 6.95 (95% CI, 5.23–9.33) for AKI cases (model 1, Figure 3), which decreased to 4.40 (95% CI, 3.22–6.05) when adjusting for more confounders (models 2 and 3) and then 3.95 (2.88–5.47) when adding surgery-related information into the models (model 4). The fully adjusted OR (model 5) was 3.69 (95% CI, 2.68–5.13). Supplementary Figure S1 shows the ROC curves of different models. The area under the ROC curve (AUC) of all models surpass 0.75 and exhibit a high degree of resemblance.

**Figure 4:**
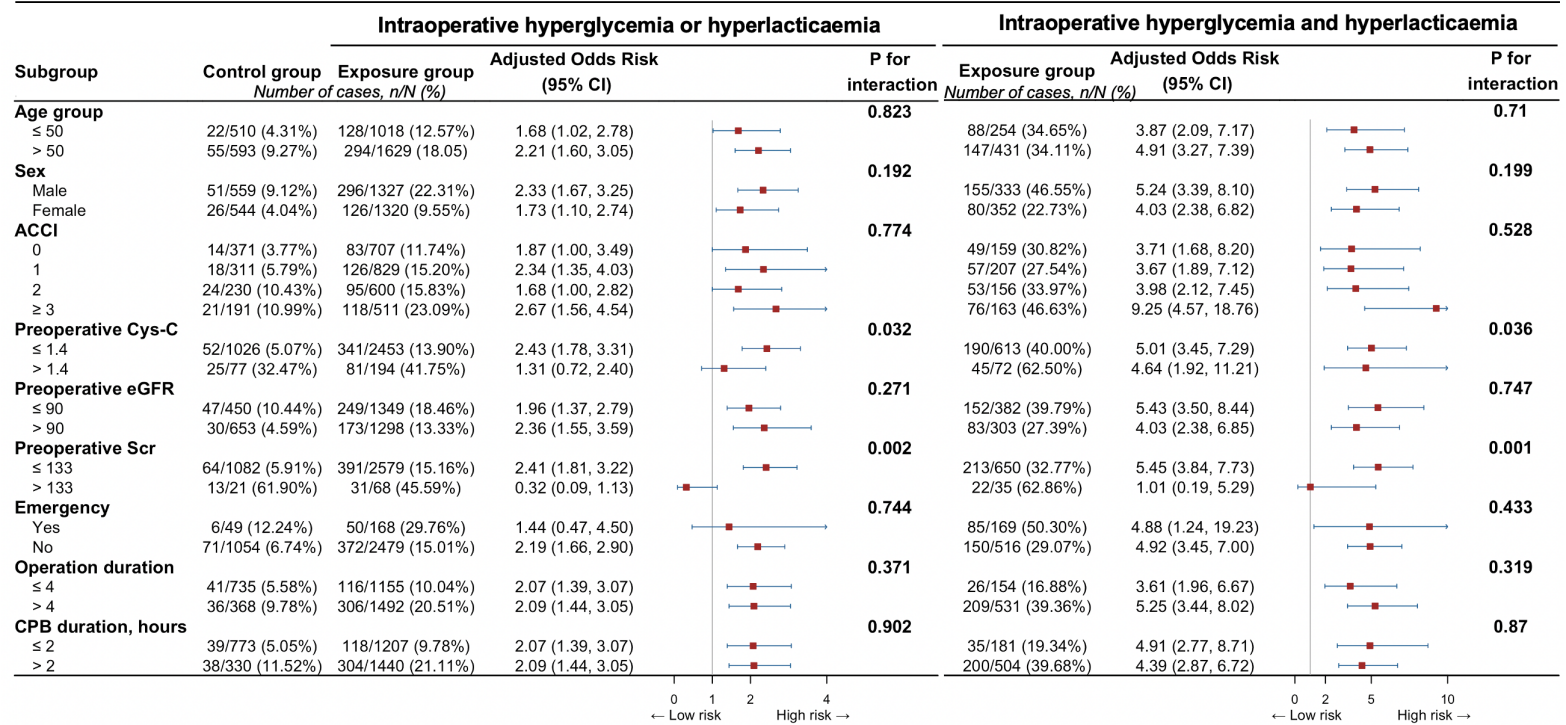
Associations between intraoperative both hyperglycemia and hyperlactatemia, or any of them, and specific subtypes of acute kidney injury. ACCI, aged-adjusted Charlson index; CI, confidence interval; Cys C, cystatin C; eGFR, estimated glomerular filtration rate; Scr, serum creatinine concentration.

Subgroup analyses indicated the observed associations could not be modified by age group, sex, ACCI, preoperative Cys-c, preoperative eGFR, preoperative Scr, emergency, operation duration, and CPB duration (Figure 4).

## 4. Discussion

### 4.1. Main findings

In this study, we aimed to evaluate the potential correlation between intraoperative hyperglycemia, hyperlactatemia, or both, and the occurrence of AKI in a series of patients undergoing on-pump cardiac surgery. After full adjustment for confounders, we found that both intraoperative hyperglycemia and hyperlactatemia (or either of them alone) were linked to a higher risk of postoperative AKI. Specifically, experiencing intraoperative hyperglycemia or hyperlactatemia was associated with approximately 2-fold the risk of developing postoperative AKI, whereas experiencing both of these conditions was associated with a 3.7-fold increased risk. These findings emphasized that managing metabolic factors properly, by avoiding intraoperative hyperglycemia or hyperlactatemia, could be beneficial for lowering AKI occurrence.

### 4.2. Previous findings

Some studies have shown that even mild, transient increases in Scr following cardiac surgery are associated with the progression of CKD and increased long-term mortality (26, 27). Therefore, it is important to identify high-risk groups accurately before the onset of AKI. In 1997, Chertow et al. established and validated the first predictive model for AKI after cardiac surgery (28). Subsequently, over the next few decades, a total of eight predictive models were developed (29). However, only a few models have included perioperative blood glucose or lactate levels as potential predictors. Subsequent studies emphasized the importance of monitoring glucose or lactate levels during the perioperative period on reducing complications (14, 30). For instance, Gandhi et al. retrospectively included 409 adult cardiac surgical patients at the Mayo Clinic, and suggested that intraoperative hyperglycemia was an independent risk factor for postoperative complications including death, AKI, urinary tract infections, etc. (17). Similar to previous studies, our research has revealed the presence of intraoperative hyperglycemia and hyperlactatemia was associated with an increased risk of AKI in on-pump cardiac surgery. Notably, the increased risk of AKI is striking when both abnormalities are present. This finding further highlighted the necessity of developing glucose/lactate intervention for postoperative kidney function management after on-pump cardiac surgery.

### 4.3. Clinical implications

Hyperglycemia intraoperatively is prevalent in over 60% of patients who undergo cardiac surgery, and is known to cause endothelial and end-organ dysfunction in the postoperative period (14). Elevated blood glucose levels can further exacerbate the inflammatory response, consistent with the pathophysiological process of AKI occurrence. However, perioperative hyperglycemia management is controversial, with conflicting results from trials assessing the potential benefits of strict glucose control (target range 80–110 mg/dl) (31). Specifically, several studies reported reduced mortality and morbidity via rigorous management of blood glucose, but subsequent studies showed a lack of benefit or even worse outcomes (32–35). Due to the uncertainty surrounding the effectiveness of different protocols targeting perioperative glycemic control, most medical societies recommend a moderate approach to glycemic control in the perioperative and intensive care setting, with patients maintaining serum glucose levels <180 mg/dl, regardless of their diabetic status (36, 37).

Nevertheless, there is an ongoing debate regarding whether there are discrepancies in the correlation between hyperglycemia and adverse postoperative outcomes in patients with and without diabetes. Ascione et al.'s study found no significant correlation between perioperative blood glucose control levels and the incidence of postoperative kidney insufficiency in diabetic patients, but an opposite result was observed in non-diabetic patients (11). In contrast, a study conducted in Italy suggested that hyperglycemia, regardless of the presence of diabetes, is an independent predictor of postoperative AKI in cardiac surgery (15). Thus, research on cut-off levels of intraoperative glucose to releasing AKI risk remains poorly investigated.

Blood lactate levels serves as a biomarker of systemic or regional hypoperfusion (38). For instance, elevated lactate levels have been identified as an independent predictor of sepsis-associated AKI (39). However, Blood lactate levels can be affected by various factors. Cold and warm ischemia times may play a major role in lactate production during heart transplantation (40). Off-pump coronary artery bypass graft surgery is associated with a reduced release of lactate, possibly due to a mechanism related to the cellular inflammatory response (41). Therefore, patients underwent heart transplantation or off-pump cardiac surgery were excluded from the current study. As a non-physiological state, CPB can interfere with tissue perfusion and lead to elevated lactate levels (16). Previous studies have revealed that 10%–26.6% of patients underwent cardiac surgery with CPB experience hyperlactatemia during or shortly after the procedure, which is strongly associated with target organ insufficiency (30, 42–44). Consequently, an increase in lactate levels may aid in predicting renal hypoperfusion and early detection of postoperative AKI.

A defined threshold for elevated lactate levels has not been universally established and may vary across studies (45). Values ranging from 2.0 to 4.0 mmol/L have been reported for predicting major complications and early mortality in cardiac surgical patients (30, 45–47). Furthermore, the Third International Consensus Definitions for Sepsis and Septic Shock have updated the criteria for sepsis and septic shock to include high blood lactate levels (>2 mmol/L) as a diagnostic indicator for septic shock (48). In present study, a cut-off value of 2.0 mmol/L was chosen arbitrarily based on common clinical practice.

Previous studies revealed a higher mortality for late postoperative hyperlactatemia, which may reflect prolonged tissue hypoperfusion or increased oxygen utilization (49). Meanwhile, some investigators suggested early hyperlactatemia is a better predictor of mortality than late hyperlactatemia (13). Our findings are consistent with our hypothesis, indicating a correlation between intraoperative hyperlactatemia and the incidence of postoperative AKI.

Lactate and glucose levels may have an interdependent relationship in the context of postoperative AKI. Elevated blood glucose triggers inflammation and oxidative stress, leading to renal tubular pathology, and lactate accumulation impairs acid-base regulation in tubules. Kidneys handle about 30% of lactate metabolism via gluconeogenesis or complete oxidation (50). In the context of lactic acidosis, this burden becomes notably more substantial. Several studies suggest that, high lactate levels are associated with poor intraoperative and postoperative glucose control (51, 52). However, some studies have suggested that under the stress-induced hyperglycemia caused by surgery, the conversion of glucose to pyruvate may eventually decrease, leading to a reduction in lactate production (53). Additional research is required for a comprehensive comprehension of these intricate physiopathological processes.

In this study, we evaluated the risk of AKI following cardiac surgery in patients with intraoperative hyperglycemia or hyperlactatemia, as well as experiencing both of these conditions. Our study revealed that patients who had either hyperlactatemia or hyperglycemia during cardiac surgery were at an increased risk of postoperative AKI. Furthermore, when both conditions were present, the risk of postoperative AKI was even significantly higher.

Therefore, combining intraoperative blood glucose and lactate concentration as the regulating index for patients undergoing cardiac surgery may reduce the incidence of AKI, and metabolic factors-guided fluid resuscitation may be one of the strategies to mitigate organ dysfunction (54). Optimizing preoperative glycemic control is needed to correct hyperglycemia to acceptable levels. Blood glucose monitoring intraoperatively, with insulin or other medication adjustments as needed is essential, all while preventing the occurrence of hypoglycemia. Preoperative lactate evaluation should be performed to screen for potential metabolic problems. Continuous intraoperative lactate monitoring, particularly in high-risk situations, is recommended. Targeted interventions, such as improving oxygen delivery and correcting metabolic acidosis, should address elevated lactate levels. Of course, fluid intake should be adjusted based on parameters like blood pressure, heart rate, and urine output, employing a balanced approach with crystalloid and colloid solutions to correct hypovolemia without excessive fluid overload. These principles should be individualized based on patient characteristics and intraoperative findings, guided by a multidisciplinary perioperative team. Timely monitoring and feedback are crucial for personalized treatment plans. Future studies need to assess possible improvement in outcomes under more precise monitoring and tighter control of perioperative hyperglycemia and hyperlactatemia.

### 4.4. Strength and limitations

Our findings provide additional support for the association between intraoperative glucose and lactate levels and postoperative AKI, and underscore the importance of implementing improved intraoperative glucose and lactate control in cardiac surgery patients. Future prospective studies should investigate whether the duration or severity of intraoperative hyperglycemia and hyperlactatemia have an impact on prognosis. Moreover, further randomized controlled trials are necessary to validate whether a moderate glucose and lactate control strategy (aiming to maintain glucose levels no more than 10.0 mmol/L and lactate levels no more than 2.0 mmol/L) can effectively reduce the incidence of postoperative AKI.

This study has several limitations. Firstly, it was conducted retrospectively, and thus is inherently subject to patient recruitment and data collection bias. Secondly, although we adjusted for many variables, the results may still be confounded by unmeasured factors. For instance, we currently lack information regarding the duration of cross-clamping. As an alternative, we utilized the CPB duration. Thirdly, the generalizability of our findings may be limited due to the complexity of on-pump cardiac surgery, as institutions may vary in their transfusion and CPB strategies, pharmacologic support, and perioperative management. Finally, the determination of optimal cut-off values for intraoperative glucose and lactate levels to prevent postoperative AKI in cardiac surgery was not performed in this study. Further tailored prospective studies will be necessary to assess perioperative blood glucose and lactate management thresholds.

## 5. Conclusion

Hyperglycemia and hyperlactatemia are common in on-pump cardiac surgical patients. Current data demonstrates an association between elevated glucose, lactate and AKI complications in on-pump cardiac surgical patients. Future studies are warranted to assess the generalizability of the current findings.

## Data Availability

The original contributions presented in the study are included in the article/**Supplementary Material**, further inquiries can be directed to the corresponding author.
