# Supplementary material for: Association between intraoperative hyperglycemia/hyperlactatemia and acute kidney injury following on-pump cardiac surgery: a retrospective cohort study
Source: Front Cardiovasc Med. 2023 Dec 8;10:1218127. doi: 10.3389/fcvm.2023.1218127 (PMC10739479; doi:10.3389/fcvm.2023.1218127)
Supplement: Supplementary file 1 [file Table1.docx]

***Supplementary Material***

**Supplementary Table S1** Collinearity analysis with variance inflation factors.

| Factors | **VIF (95% CI)** | **SE** | **Tolerance (95% CI)** |
| --- | --- | --- | --- |
| Age | 3.77 (3.58, 3.99) | 1.94 | 0.26 (0.25, 0.28) |
| Sex | 2.04 (1.95, 2.14) | 1.43 | 0.49 (0.47, 0.51) |
| BMI | 1.15 (1.11, 1.19) | 1.07 | 0.87 (0.84, 0.90) |
| ACCI | 3.24 (3.07, 3.42) | 1.80 | 0.31 (0.29, 0.33) |
| ASA classification | 1.76 (1.68, 1.84) | 1.33 | 0.57 (0.54, 0.59) |
| NYHA | 1.20 (1.16, 1.25) | 1.10 | 0.83 (0.80, 0.86) |
| EuroSCORE II | 3.80 (3.60, 4.02) | 1.95 | 0.26 (0.25, 0.28) |
| LVEF | 1.28 (1.23, 1.33) | 1.13 | 0.78 (0.75, 0.81) |
| Diabetes mellitus | 1.20 (1.16, 1.25) | 1.10 | 0.83 (0.80, 0.86) |
| CHD | 2.15 (2.05, 2.26) | 1.47 | 0.46 (0.44, 0.49) |
| COPD | 1.09 (1.06, 1.14) | 1.05 | 0.91 (0.88, 0.94) |
| Unstable angina | 1.51 (1.45, 1.58) | 1.23 | 0.66 (0.63, 0.69) |
| Recent myocardial infarct | 1.64 (1.57, 1.72) | 1.28 | 0.61 (0.58, 0.64) |
| Pulmonary hypertension | 1.25 (1.20, 1.30) | 1.12 | 0.80 (0.77, 0.83) |
| Previous cardiac surgery | 1.51 (1.45, 1.58) | 1.23 | 0.66 (0.63, 0.69) |
| Emergency | 2.45 (2.33, 2.58) | 1.57 | 0.41 (0.39, 0.43) |
| Anesthesia | 1.09 (1.06, 1.14) | 1.05 | 0.92 (0.88, 0.94) |
| Operation duration | 2.12 (2.02, 2.23) | 1.46 | 0.47 (0.45, 0.50) |
| CPB duration | 2.08 (1.99, 2.19) | 1.44 | 0.48 (0.46, 0.50) |
| Operation type | 6.31 (5.95, 6.68) | 2.51 | 0.16 (0.15, 0.17) |
| Preoperative HB | 1.66 (1.59, 1.74) | 1.29 | 0.60 (0.58, 0.63) |
| Preoperative NT-pro BNP | 1.50 (1.44, 1.56) | 1.22 | 0.67 (0.64, 0.70) |
| Preoperative cTnT | 1.07 (1.04, 1.11) | 1.03 | 0.94 (0.90, 0.96) |
| Preoperative Cys C | 2.78 (2.64, 2.93) | 1.67 | 0.36 (0.34, 0.38) |
| Preoperative eGFR | 7.16 (6.75, 7.59) | 2.67 | 0.14 (0.13, 0.15) |
| Preoperative albumin | 1.51 (1.45, 1.58) | 1.23 | 0.66 (0.63, 0.69) |
| Preoperative Scr | 7.40 (6.98, 7.85) | 2.72 | 0.14 (0.13, 0.14) |
| RBC infusion | 1.34 (1.29, 1.40) | 1.16 | 0.74 (0.71, 0.77) |
| Plasma infusion | 1.34 (1.30, 1.40) | 1.16 | 0.74 (0.71, 0.77) |
| Cryoprecipitate infusion | 1.04 (1.02, 1.09) | 1.02 | 0.96 (0.92, 0.98) |
| Platelet infusion | 1.72 (1.65, 1.80) | 1.31 | 0.58 (0.56, 0.61) |
| Autotransfusion | 1.22 (1.18, 1.27) | 1.11 | 0.82 (0.78, 0.85) |
| Crystalloid | 1.21 (1.17, 1.26) | 1.10 | 0.83 (0.79, 0.86) |
| Colloid | 1.08 (1.05, 1.12) | 1.04 | 0.93 (0.89, 0.95) |
| Urinary | 1.19 (1.15, 1.24) | 1.09 | 0.84 (0.81, 0.87) |
| Epinephrine | 1.08 (1.05, 1.13) | 1.04 | 0.93 (0.89, 0.95) |
| Norepinephrine | 1.18 (1.15, 1.23) | 1.09 | 0.84 (0.81, 0.87) |
| Millinon | 1.16 (1.12, 1.21) | 1.08 | 0.86 (0.83, 0.89) |
| Dopamine | 1.04 (1.02, 1.09) | 1.02 | 0.96 (0.92, 0.98) |
| Nitroglycerin | 1.13 (1.09, 1.17) | 1.06 | 0.89 (0.85, 0.91) |
| Aspirin | 1.44 (1.39, 1.51) | 1.20 | 0.69 (0.66, 0.72) |
| β-blockers | 1.16 (1.12, 1.20) | 1.08 | 0.86 (0.83, 0.89) |
| LMWH | 1.25 (1.21, 1.30) | 1.12 | 0.80 (0.77, 0.83) |

Abbreviation: ACCI, age-adjusted Charlson Comorbidity Index; ASA, American Society of Anesthesiologists classification; BMI, body-mass index; CHD, congenital heart disease; CI, confidence interval; COPD, chronic obstructive pulmonary disease; CPB, cardiopulmonary bypass; cTnT, Cardiac troponin T; Cys C, Cystatin C; EuroSCORE II, European system for cardiac operative risk evaluation II; HB, hemoglobin; LVEF, Left Ventricular Ejection Fractions; LMWH, low molecular weight heparin; NYHA, New York Heart Association Classification; NT-pro BNP, N-terminal pro-brain natriuretic peptide; RBC, red blood cell; Scr, serum creatinine concentration; SE, standard error; VIF, variance inflation factor.

**Supplementary Table S2** Outcome stratified by different exposure.

|  | **Total** | **Non-Intraoperative hyperglycemia or hyperlacticaemia** | **Intraoperative hyperglycemia or hyperlacticaemia** | **Intraoperative hyperglycemia and hyperlacticaemia** | ***P*-value** |
| --- | --- | --- | --- | --- | --- |
| No. of patients | 4435 | 1103 | 2647 | 685 |  |
| **Outcome, n (%)** |  |  |  |  |  |
| AKI |  |  |  |  | <0.001 ^a^ |
| Postoperative non-AKI | 3701 (83.45) | 1026 (93.02) | 2225 (84.06) | 450 (65.69) |  |
| Postoperative AKI | 734 (16.55) | 77 (6.98) | 422 (15.94) | 235 (34.31) |  |
| AKI severity**^*^** |  |  |  |  |  |
| Stage 1 | 581 (13.10) | 66 (5.98) | 355 (13.41) | 160 (23.36) |  |
| Stage 2 | 84 (1.89) | 4 (0.36) | 40 (1.51) | 40 (5.84) |  |
| Stage 3 | 69 (1.56) | 7 (0.63) | 27 (1.02) | 35 (5.11) |  |

Data are present in n (%), mean (SD) or median (IQR).

P-values are derived from: ^a^ chi-square test.

**^*^**Stage 1 AKI, creatinine rise 0.3 mg/dl or greater within 48 h or 1.5 to 1.9 times baseline within first 7-day after surgery; stage 2 AKI, creatinine rise 2.0 to 2.9 times baseline within 7-day after surgery; and stage 3 AKI, creatinine rise to 4.0 mg/dl or greater or 3.0 times baseline.

Abbreviation: AKI, acute kidney injury.

**Supplementary Table S3** Demographics, clinical characteristics, and perioperative data stratified by different exposure.

|  | **Non-Intraoperative hyperglycemia or hyperlacticaemia** | **Intraoperative hyperglycemia or hyperlacticaemia** | **Intraoperative hyperglycemia and hyperlacticaemia** | ***P*-value** |
| --- | --- | --- | --- | --- |
| No. of patients | 1103 | 2647 | 685 |  |
| **Demographic characteristics** |  |  |  |  |
| Age, years, median (IQR) | 52.00 (41.00, 59.00) | 53.00 (47.00, 61.00) | 54.00 (47.00, 62.00) | <0.001 ^a^ |
| Age, years, n (%) |  |  |  | <0.001 ^b^ |
| ≤ 50 | 510 (46.24) | 1018 (38.46) | 254 (37.08) |  |
| > 50 | 593 (53.76) | 1629 (61.54) | 431 (62.92) |  |
| Sex, n (%) |  |  |  | 0.688 ^b^ |
| Male | 559 (50.68) | 1327 (50.13) | 333 (48.61) |  |
| Female | 544 (49.32) | 1320 (49.87) | 352 (51.39) |  |
| BMI, kg/m^2^, median (IQR) | 22.49 (20.62, 24.75) | 23.33 (21.23, 25.56) | 24.23 (21.98, 26.67) | <0.001 ^a^ |
| **Preoperative comorbidities** |  |  |  |  |
| ACCI, n (%) |  |  |  | <0.001 ^b^ |
| 0 | 371 (33.64) | 707 (26.71) | 159 (23.21) |  |
| 1 | 311 (28.20) | 829 (31.32) | 207 (30.22) |  |
| 2 | 230 (20.85) | 600 (22.67) | 156 (22.77) |  |
| ≥ 3 | 191 (17.32) | 511 (19.30) | 163 (23.80) |  |
| ASA classification, n (%) |  |  |  | <0.001 ^b^ |
| < 3 | 1059 (96.01) | 2436 (92.03) | 491 (71.68) |  |
| ≥ 3 | 44 (3.99) | 211 (7.97) | 194 (28.32) |  |
| NYHA, n (%) |  |  |  | <0.001 ^b^ |
| 1 | 115 (10.43) | 156 (5.89) | 28 (4.09) |  |
| 2 | 636 (57.66) | 1440 (54.40) | 391 (57.08) |  |
| 3 | 331 (30.01) | 993 (37.51) | 248 (36.20) |  |
| 4 | 21 (1.90) | 58 (2.19) | 18 (2.63) |  |
| EuroSCORE II, median (IQR) | 1.00 (0.00, 3.00) | 2.00 (1.00, 4.00) | 4.00 (1.00, 7.00) | <0.001 ^a^ |
| LVEF, n (%) |  |  |  | <0.001 ^b^ |
| ≥ 50% | 1028 (93.20) | 2369 (89.50) | 606 (88.47) |  |
| 30 ~ 49% | 69 (6.26) | 258 (9.75) | 76 (11.09) |  |
| < 30% | 6 (0.54) | 20 (0.76) | 3 (0.44) |  |
| Diabetes mellitus, n (%) | 78 (7.07) | 110 (4.16) | 70 (10.22) | <0.001 ^b^ |
| CHD, n (%) | 102 (9.25) | 213 (8.05) | 59 (8.61) | 0.475 ^b^ |
| COPD, n (%) | 48 (4.35) | 111 (4.19) | 42 (6.13) | 0.089 ^b^ |
| Unstable angina, n (%) | 18 (1.63) | 45 (1.70) | 13 (1.90) | 0.912 ^b^ |
| Recent myocardial infarct**^†^**, n (%) | 17 (1.54) | 29 (1.10) | 18 (2.63) | 0.011 ^b^ |
| Pulmonary hypertension**^‡^**, n (%) |  |  |  | 0.458 ^b^ |
| None | 1040 (94.29) | 2497 (94.33) | 638 (93.14) |  |
| Moderate | 50 (4.53) | 124 (4.68) | 42 (6.13) |  |
| Severe | 13 (1.18) | 26 (0.98) | 5 (0.73) |  |
| Previous cardiac surgery, n (%) | 58 (5.26) | 203 (7.67) | 72 (10.51) | <0.001 ^b^ |
| **Operative data** |  |  |  |  |
| Emergency, n (%) | 49 (4.44) | 168 (6.35) | 169 (24.67) | <0.001 ^b^ |
| Anesthesia, n (%) |  |  |  | <0.001 ^b^ |
| Intravenous and inhalation | 940 (85.22) | 2264 (85.53) | 620 (90.51) |  |
| Total intravenous | 119 (10.79) | 255 (9.63) | 31 (4.53) |  |
| Inhalation | 44 (3.99) | 128 (4.84) | 34 (4.96) |  |
| Operation duration, min, median (IQR) | 216.50 (180.00, 257.00) | 252.00 (214.00, 306.00) | 320.50 (247.75, 423.00) | <0.001 ^a^ |
| Operation duration, hours, n (%) |  |  |  | <0.001 ^b^ |
| ≤ 4 | 735 (66.64) | 1155 (43.63) | 154 (22.48) |  |
| > 4 | 368 (33.36) | 1492 (56.37) | 531 (77.52) |  |
| CPB duration, min, median (IQR) | 97.00 (74.00, 128.00) | 126.00 (97.00, 163.00) | 163.00 (118.00, 214.00) | <0.001 ^a^ |
| CPB duration, hours, n (%) |  |  |  | <0.001 ^b^ |
| ≤ 2 | 773 (70.08) | 1207 (45.60) | 181 (26.42) |  |
| > 2 | 330 (29.92) | 1440 (54.40) | 504 (73.58) |  |
| Operation type, n (%) |  |  |  | <0.001 ^b^ |
| CABG | 75 (6.80) | 143 (5.40) | 48 (7.01) |  |
| CHD | 141 (12.78) | 139 (5.25) | 11 (1.61) |  |
| Single valve | 340 (30.83) | 685 (25.88) | 132 (19.27) |  |
| Multi valve | 47 (4.26) | 128 (4.84) | 23 (3.36) |  |
| Vascular | 77 (6.98) | 338 (12.77) | 188 (27.45) |  |
| Mixed**^*^** | 296 (26.84) | 1050 (39.67) | 248 (36.20) |  |
| Others**^**^** | 127 (11.51) | 164 (6.20) | 35 (5.11) |  |
| **Preoperative laboratory examination** |  |  |  |  |
| Preoperative HB, g/dL, median (IQR) | 134.00 (121.00, 146.00) | 136.00 (124.00, 148.00) | 132.00 (120.00, 143.00) | <0.00 ^a^ |
| Preoperative NT-pro BNP, pg/ml, median (IQR) | 313.00 (96.00, 1107.00) | 565.00 (146.00, 1436.50) | 611.00 (194.75, 1482.25) | <0.00 ^a^ |
| Preoperative cTnT, ug/L, median (IQR) | 9.50 (6.10, 16.40) | 10.45 (7.20, 17.00) | 12.70 (8.47, 23.33) | <0.00 ^a^ |
| Preoperative Cys C, mg/L, median (IQR) | 0.95 (0.84, 1.10) | 0.99 (0.87, 1.14) | 1.03 (0.87, 1.18) | <0.00 ^a^ |
| Preoperative Cys C, mg/L, n (%) |  |  |  | 0.012 ^b^ |
| ≤ 1.4 | 1026 (93.02) | 2453 (92.67) | 613 (89.49) |  |
| > 1.4 | 77 (6.98) | 194 (7.33) | 72 (10.51) |  |
| Preoperative eGFR, mL/min/1.73m^2^, median (IQR) | 94.80 (80.65, 106.12) | 89.47 (75.37, 100.58) | 86.80 (71.08, 99.66) | <0.00 ^a^ |
| Preoperative eGFR, mL/min/1.73m^2^, n (%) |  |  |  | <0.001 ^b^ |
| ≤ 90 | 450 (40.80) | 1349 (50.96) | 382 (55.77) |  |
| > 90 | 653 (59.20) | 1298 (49.04) | 303 (44.23) |  |
| Preoperative albumin, g/L, median (IQR) | 43.10 (40.40, 45.80) | 43.20 (40.60, 45.80) | 42.10 (39.20, 45.20) | <0.00 ^a^ |
| Preoperative Scr, umol/L, median (IQR) | 74.00 (63.00, 86.00) | 76.00 (65.00, 89.00) | 77.00 (65.00, 91.00) | <0.00 ^a^ |
| Preoperative Scr, umol/L, n (%) |  |  |  | <0.001 ^b^ |
| ≤ 133 | 1082 (98.10) | 2579 (97.43) | 650 (94.89) |  |
| > 133 | 21 (1.90) | 68 (2.57) | 35 (5.11) |  |
| **Intraoperative fluid management** |  |  |  |  |
| RBC infusion, n (%) | 74 (6.71) | 234 (8.84) | 155 (22.63) | <0.001 ^b^ |
| Plasma infusion, n (%) | 51 (4.62) | 232 (8.76) | 146 (21.31) | <0.001 ^b^ |
| Cryoprecipitate infusion, n (%) | 1 (0.09) | 16 (0.60) | 7 (1.02) | 0.014 ^b^ |
| Platelet infusion, n (%) | 228 (20.67) | 768 (29.01) | 380 (55.47) | <0.001 ^b^ |
| Autotransfusion, mL, median (IQR) | 300.00 (250.00, 400.00) | 300.00 (250.00, 500.00) | 400.00 (300.00, 600.00) | <0.001 ^a^ |
| Crystalloid, mL, median (IQR) | 700.00 (400.00, 900.00) | 750.00 (500.00, 1000.00) | 850.00 (600.00, 1150.00) | <0.001 ^a^ |
| Colloid, mL, median (IQR) | 0.00 (0.00, 100.00) | 0.00 (0.00, 100.00) | 0.00 (0.00, 300.00) | <0.001 ^a^ |
| Urinary, mL, median (IQR) | 800.00 (500.00, 1200.00) | 950.00 (600.00, 1400.00) | 900.00 (600.00, 1400.00) | <0.001 ^a^ |
| **Intraoperative vasoactive agents** |  |  |  |  |
| Epinephrine, n (%) | 819 (74.25) | 2426 (91.65) | 636 (92.85) | <0.001 ^b^ |
| Total epinephrine, μg, median (IQR) | 67.70 (0.00, 173.40) | 202.30 (90.00, 317.25) | 284.20 (156.80, 455.50) | <0.001 ^a^ |
| Norepinephrine, n (%) | 239 (21.67) | 639 (24.14) | 277 (40.44) | <0.001 ^b^ |
| Total norepinephrine, μg, median (IQR) | 0.00 (0.00, 0.00) | 0.00 (0.00, 0.00) | 0.00 (0.00, 0.20) | <0.001 ^a^ |
| Millinon, n (%) | 65 (5.89) | 298 (11.26) | 95 (13.87) | <0.001 ^b^ |
| Total millinon, mg, median (IQR) | 0.00 (0.00, 0.00) | 0.00 (0.00, 0.00) | 0.00 (0.00, 0.00) | <0.001 ^a^ |
| Dopamine, n (%) | 5 (0.45) | 12 (0.45) | 4 (0.58) | 0.865 ^b^ |
| Total dopamine, mg, median (IQR) | 0.00 (0.00, 0.00) | 0.00 (0.00, 0.00) | 0.00 (0.00, 0.00) | 0.899 ^a^ |
| Nitroglycerin, n (%) | 747 (67.72) | 1893 (71.51) | 442 (64.53) | 0.001 ^b^ |
| Total nitroglycerin, mg, median (IQR) | 722.40 (0.00, 2573.40) | 1030.50 (0.00, 3347.00) | 652.80 (0.00, 3060.00) | <0.001 ^a^ |
| **Coronary heart disease related drugs** |  |  |  |  |
| Aspirin, n (%) | 57 (5.87) | 98 (4.42) | 26 (4.55) | 0.202 ^b^ |
| β-blockers, n (%) | 123 (11.15) | 368 (13.90) | 116 (16.93) | 0.002 ^b^ |
| LMWH, n (%) | 60 (5.44) | 154 (5.82) | 37 (5.40) | 0.857 ^b^ |

Data are present in n (%), mean (SD) or median (IQR).

P-values are derived from: ^a^ u-test, ^b^ chi-square test or Fisher exact test.

**^†^**Recent myocardial infarction is defined as any diagnosed myocardial infarction in past 90 days before the operation.

**^‡^**Moderate pulmonary hypertension is defined as pulmonary artery systolic pressure 31 to 55mmHg, and severe pulmonary hypertension as more than 55 mmHg.

**^*^**Mixed operations are referred to operations combined with CABG, and/or valve, and/or CHD, and/or others.

**^**^**Others operations are referred to operations that could not be included in the types above (i.e., cardiac myxoma, pulmonary endarterectomy, etc.)

Abbreviation: ACCI, age-adjusted Charlson Comorbidity Index; ASA, American Society of Anesthesiologists classification; BMI, body-mass index; CABG, coronary artery bypass grafting; CHD, congenital heart disease; COPD, chronic obstructive pulmonary disease; CPB, cardiopulmonary bypass; cTnT, Cardiac troponin T; Cys C, Cystatin C; EuroSCORE II, European system for cardiac operative risk evaluation II; HB, hemoglobin; IQR, interquartile range; LVEF, Left Ventricular Ejection Fractions; LMWH, low molecular weight heparin; NYHA, New York Heart Association Classification; NT-pro BNP, N-terminal pro-brain natriuretic peptide; RBC, red blood cell; Scr, serum creatinine concentration; SD, standard deviation.


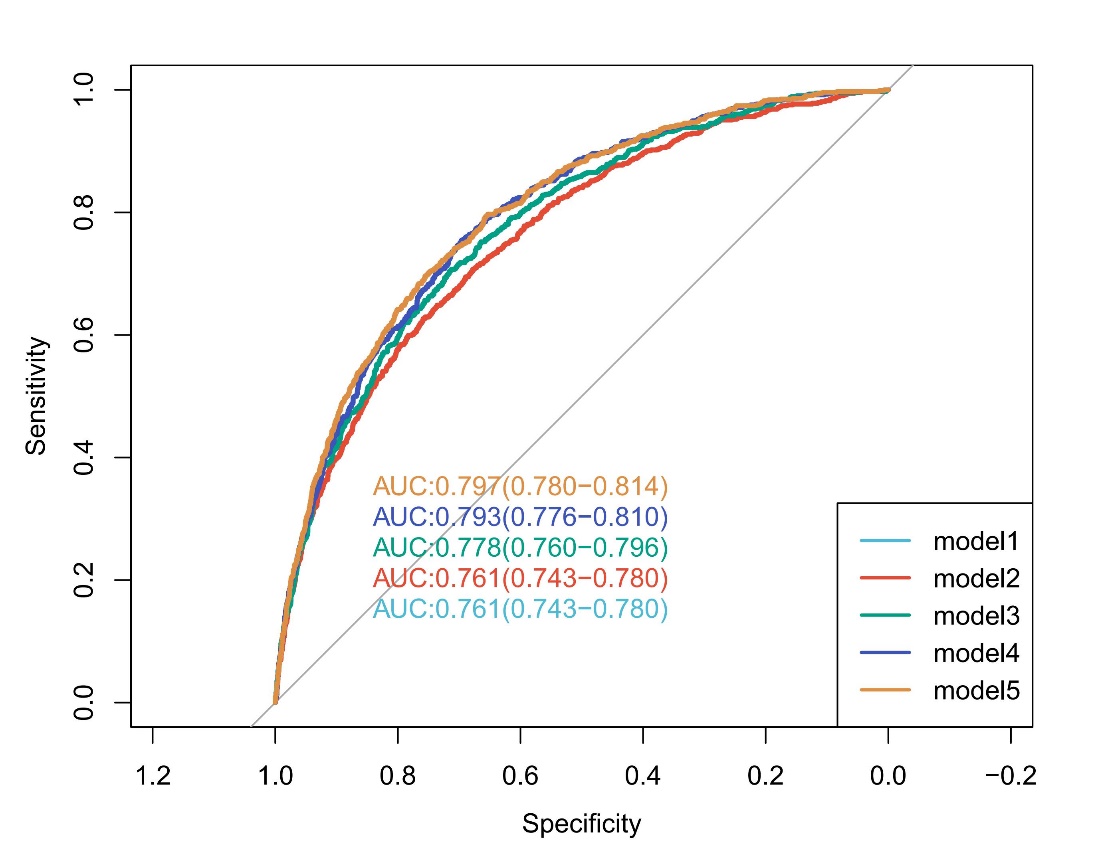


**Supplementary Figure S1** The receiver operating characteristic (ROC) plot of different models.
